# Design and Characterization of Deformable Superstructures Based on Amine‐Acrylate Liquid Crystal Elastomers

**DOI:** 10.1002/advs.202303594

**Published:** 2023-11-09

**Authors:** Fang Zhao, Yuzhan Li, Hong Gao, Ran Tao, Yiqi Mao, Yu Chen, Sheng Zhou, Jianming Zhao, Dong Wang

**Affiliations:** ^1^ Division of Material Engineering China Academy of Space Technology Beijing 100094 P. R. China; ^2^ Department of Materials Physics and Chemistry School of Materials Science and Engineering University of Science and Technology Beijing Beijing 100083 P. R. China; ^3^ Institute of Advanced Structure Technology Beijing Institute of Technology Beijing 100081 P. R. China; ^4^ Department of engineering mechanics College of Mechanical and Vehicle Engineering Hunan University Changsha Hunan 410082 P. R. China

**Keywords:** amine‐acrylate liquid crystal elastomers, deformable superstructures, finite element analyses, self‐healing capabilities, shape memory

## Abstract

Deformable superstructures are man‐made materials with large deformation properties that surpass those of natural materials. However, traditional deformable superstructures generally use conventional materials as substrates, limiting their applications in multi‐mode reconfigurable robots and space‐expandable morphing structures. In this work, amine‐acrylate‐based liquid crystal elastomers (LCEs) are used as deformable superstructures substrate to provide high driving stress and strain. By changing the molar ratio of amine to acrylate, the thermal and mechanical properties of the LCEs are modified. The LCE with *a ratio of* 0.9 exhibited improved polymerization degree, elongation at break, and toughness. Besides an anisotropic finite deformation model based on hyperelastic theory is developed for the LCEs to capture the configuration variation under temperature activation. Built upon these findings, an LCE‐based paper‐cutting structure with negative Poisson's ratio and a 2D lattice superstructure model are combined, processed, and molded by laser cutting. The developed superstructure is pre‐programmed to the configuration required for service conditions, and the deformation processes are analyzed using both experimental and finite element methods. This study is expected to advance the application of deformable superstructures and LCEs in the fields of defense and military, aerospace, and bionic robotics.

## Graphical abstract

Liquid crystal elastomers (LCEs) based on amine‐acrylate chemistry are utilized as the substrate for the development of deformable superstructures. High driving stress and strain are achieved by optimizing the molar ratio of amine and acrylate. Built upon finite element simulation, origami‐inspired and 2D lattice superstructures are developed, exhibiting reversible and controlled deformations.

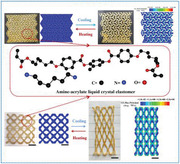

## 1 Introduction

With the advances of science and technology, deformable superstructures have become a research area of great interest. They are artificially designed periodic or non‐periodic materials with large deformation properties and mechanical functions beyond those of natural materials. They have broad application prospects in national defense, military, aerospace, biomedicine, etc. A variety of practical reconfigurable electromagnetic devices (systems) have been developed using deformable superstructures,^[^ ^1^ ^]^ including variable curvature wings,^[^ ^2^ ^]^ biaxial thermal‐shrinking promoting wound‐healing dressings.^[^ ^3^, ^4^ ^]^ The unique multiscale structural configuration of metamaterials exhibits unconventional deformation behaviors, such as negative Poisson's ratio,^[^ ^5^, ^6^ ^]^ negative stiffness,^[^ ^7^, ^8^ ^]^ compression‐torsion coupling response,^[^ ^9^ ^]^ and negative thermal expansion,^[^ ^10^, ^11^ ^]^ exhibiting potential applications in flexible components and smart robots.^[^ ^12^, ^13^, ^14^ ^]^ However, traditional reconfigurable metamaterials generally use conventional materials (steel, elastoplastic, silicone elastomer, and natural latex rubber^[^ ^15^, ^16^ ^]^) as substrates, which lack self‐healing capabilities and exhibit problems in actuation and shape regulation after synthesis. A potential solution is the use of liquid crystalline elastomers (LCEs) because of its stimulus responsiveness, shape memory effect, and self‐healing ability.

LCEs are a class of liquid crystalline (LC) polymers that possess LC order with moderate cross‐linking, which was first proposed by de Gennes.^[^ ^17^ ^]^ LCEs exhibit both the elasticity of elastomers and the fluidity and order of liquid crystals. The change in the degree of order of liquid crystal elements is the intrinsic driving force behind large deformations produced when LCEs are exposed to heat,^[^ ^4^, ^18^ ^]^ light,^[^ ^19^, ^20^, ^21^, ^22^ ^]^ electricity,^[^ ^23^, ^24^ ^]^ humidity,^[^ ^25^ ^]^ and other external stimuli (pH‐driven LCEs^[^ ^26^, ^27^ ^]^). After the concept of LCE was conceived, Finkelmann pioneered a two‐stage hydrosilylation method for the synthesis of the world's first independent‐driven “nematic liquid single crystal elastomer” in 1991 using silane hydrides and alkenes to introduce an asymmetric crosslinking agent.^[^ ^28^ ^]^ Later, Donnio et al.^[^ ^29^ ^]^ improved Finkelmann's two‐stage hydrosilylation method and proposed a one‐pot method for the water‐silane reaction between a diethylene‐reactive intermediate and a disiloxane chain‐extender and a tetrasiloxane crosslinker. In recent years, the main method of LCE synthesis is the thiol‐acrylate and acrylate‐acrylate two‐stage addition method reported by Yakachi et al.^[^ ^30^ ^]^ in 2015, which improved the stability of the intermediate product compared to the previously reported two‐stage synthesis. However, it has some disadvantages such as poor irradiation depth and limited sample size due to the second‐stage UV curing process. Therefore, for the preparation of deformable superstructures, it is particularly important to select LCEs with easy fabrication and better overall mechanical properties. Compared with similar elastomers, this amine‐acrylate system LCEs is not only simple to use and has a long window period, but also significantly improved in mechanical properties. The elongation at break of ordinary thiol‐acrylate LCEs is generally lower than 200%, while the elongation at break of the present LCEs reaches more than 400%, and the breaking stress is elevated to 34 MPa compared to the 0.5–1.4 MPa of ordinary LCEs.^[^ ^31^, ^32^, ^33^, ^34^ ^]^ This study improves the comprehensive performance of LCEs and expands their possible applications.

To model and simulate the bending and curling characteristics of LCE beams, Potekhina and Wang^[^ ^35^ ^]^ developed a simple layered 2D model based on the gradient of the temperature‐dependent equivalent thermal expansion. By measuring the radius of curvature of the LCE film aligned unidirectionally at one surface produced on a rubbed Kapton film, the appropriate parameters were derived. Neufeld et al.^[^ ^36^ ^]^ presented a method using a hyperelastic solid mechanics model and experimental measurement of material parameters for a thermotropic LCE. In order to determine the optimal design parameters for gripper performance, the simulation method is used to perform a proof‐of‐concept design process of an LCE multi‐legged gripper. Through rationally designed experiments guided by simulations, Zhang et al.^[^ ^37^ ^]^ explored the optimal formulation of a LCE artificial muscle and established the relationship between shape transformation behaviors and geometrical parameters of the kirigami structures. Wu et al.^[^ ^38^ ^]^ introduced a triaxial order tensor to describe the complex alignment under uniaxial/biaxial loading conditions for establishing a constitutive model of LCE, and proposed an equivalent order parameter to characterize the degree of alignment inspired by the classical theory of plasticity. Wang et al.^[^ ^39^ ^]^ developed a theory for the large deformation viscoelastic behavior of monodomain nematic elastomers that separately incorporate the hypothesized dissipation mechanisms of viscous mesogen rotation and viscoelastic network deformation.

In this paper, we studied the preparation and simulation methods of amine‐acrylate‐based LCE for the development of deformable superstructures. The prepared LCE exhibited excellent performance and was used for establishing its large deformation intrinsic model. The combined experimental and finite element analysis methods allowed for a mutual verification of the superstructure deformation process. This study fills the current lack of technology by combining the preparation method and design analysis of LCE‐based deformable superstructure, and lays the foundation for realizing the application in the fields of bionic robots and flexible parts.

## 2 Results and Discussion

### 2.1 Preparation and Characterization of Amine‐Acrylate LCE

**Scheme 1:**
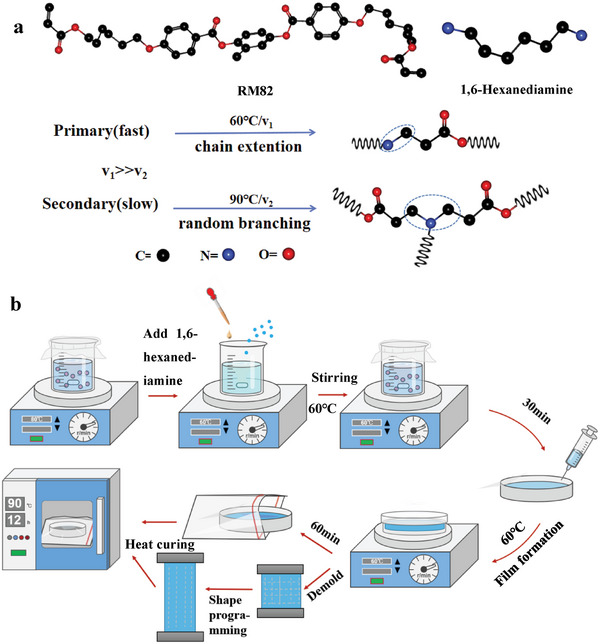
Composition and reaction of amine‐acrylate LCE. a) Amine‐acrylate LCEs reaction mechanism. b) Schematic illustration of LCEs film preparation process.

In this paper, LCE based on amine‐acrylate Michael addition was selected as the deformable superstructure substrate. In the first stage, the primary amine of 1,6‐hexanediamine acts as a chain extender similar to a dithiol, resulting in a rapid thermal curing process. The reaction rate is denoted as v_1_. After film formation, the LCE prepolymer can be deformed into the desired shape through shape programming. The mechanical orientation process causes the liquid crystal units within the LCE to align along the programming direction, resulting in a fixed molecular orientation. In the second stage, the LCE undergoes a 12 h thermal curing process at 90 °C, during which the remaining ‐H on the diamine reacts with the C═C bonds in excess acrylate. The reaction rate is denoted as v_2_. The formation mechanism relies on the molecular kinetic difference, where v_1_ >> v_2_^[^ ^35^, ^40^, ^41^, ^42^ ^]^ (**Scheme** **1**a). LCE prepared in this manner exhibits a two‐way shape memory effect, where the liquid crystal molecules undergo a phase transition to an isotropic state upon heating and reorganize into an anisotropic state upon cooling.

**Figure 1:**
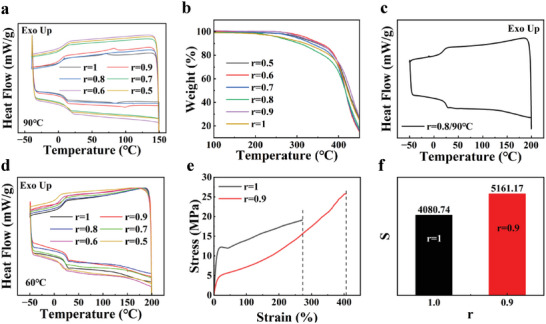
LCEs with different diamine/diacrylate molar ratios and the corresponding mechanical properties. a) DSC plots of LCEs with r = 0.5–1 after the second stage of thermal curing. b) Thermogravimetry analysis (TGA) of LCEs with r = 0.5–1. c) DSC plots of LCE _r = 0.8_ after the completion of the second stage thermal curing. d) DSC plots of LCE with r = 0.5–1 after the first stage of thermal curing. e) Tensile stress‐strain curves of LCE for r = 1 and r = 0.9. f) LCE toughness for r = 1 and r = 0.9 obtained by integration of the stress‐strain curves for e).

Theoretically, it requires a molar ratio of diamine/diacrylate (r) less than 1 for gelation.^[^ ^42^ ^]^ To explore the suitable crosslinking density for actuation, LCEs with r = 0.5–1 (Denoted as LCE _r=0.5‐1_)were prepared and differential scanning calorimetry (DSC) tests were performed (**Figure** **1**a). The results showed that the phase transition temperature (T_NI_) of LCE disappeared when r ≤ 0.8, and only the phase transition temperatures of LCE _r = 1_ and LCE _r = 0.9_ were detected, which were 70.5 °C and 82.6 °C, respectively. For r ≤ 0.8, the T_NI_ of LCE disappeared due to the increased cross‐link density. In order to test the thermal stability of the LCEs, thermogravimetry analysis (TGA) tests were performed on the fully cured LCEs, and the results showed that the LCEs were thermally stable up to 250 °C (Figure 1b). The derivative thermogravimetry (DTG) test results are presented in Figure S1 (Supporting Information). Therefore, in order to investigate whether the temperature during the DSC test affects the T_NI_, the temperature range of the DSC test was extended to −50–200 °C. However, the DSC test results still did not find the phase transition temperature of *r* = 0.8 (Figure 1c). The DSC tests of the first‐stage partially cured (60 °C) LCEs are shown in Figure 1d, and T_NI_ was not observed for LCE with *r* ≤ 0.8. It was further confirmed by polarizing microscope (POM) that the fully cured LCE _r = 0.8_ film showed no obvious brightness change before and after a rotation of 45° (Figure S2, Supporting Information), indicating that the mesogen within LCE _r = 0.8_ had no obvious alignment and was not suitable to be used as actuating materials. This is in contrast to the POM image with LCE _r = 0.9_ (Figure S3, Supporting Information). The pre‐stretched LCE _r = 0.7–0.5_ either broke directly during the stretching process, or broke during the second‐stage of heat curing, with high stiffness and poor toughness. It was concluded that for r ≤ 0.8 the self‐polymerization reaction of acrylate competed with the addition reaction between acrylate and amine, while for the LCEs with r values of 0.9 and 1 the overall reaction was dominated by the addition between amine and acrylate, resulting in a more homogeneous network structure and therefore the T_NI_ could be observed. Moreover, the T_NI_ was observed more clearly after the completion of the second‐stage of thermal curing.

In order to select the best r, mechanical properties of the LCEs with r = 0.9 and r = 1 ratios were tested. The results showed that the breaking elongation(Figure 1e) and toughness(Figure 1f) of the LCE with r = 0.9 were better than those of the LCE with r = 1. Therefore, the LCE formulation of r = 0.9 was selected for the following work to ensure optimal performance and uniformity.

**Figure 2:**
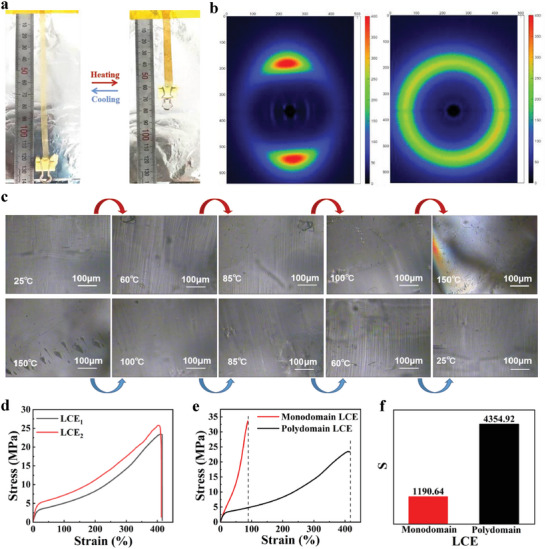
Characterization of actuation properties of LCE based on amine‐acrylate reaction. a) Reversible deformation of fully cured LCE films during a heating‐cooling cycle. b) X‐ray diffraction pattern of monodomain and polydomain LCEs. c) POM images of reversible deformation of uniaxially aligned LCE film during heating and cooling cycles. d) Tensile stress‐strain curves of polydomain LCE tested in perpendicular directions. e) Stress‐strain curves for polydomain and monodomain LCE. f) Monodomain and polydomain LCE toughness obtained by integrating the stress‐strain curves of e).

Upon gelation, the partially cured gel exhibited robust mechanical properties and could be easily stretched to over 400% at room temperature (Figure S4, Supporting Information). The stretched LCE was heated to 90 °C for 12 h to facilitate the cross‐linking. The resulting monodomain LC alignment was confirmed by its clear transparent appearance, POM characterization, X‐ray characterization, and fully reversible actuation upon temperature cycling (**Figure** **2**a). The 2D X‐ray scattering (2D‐WAXS) images (Figure 2b) showed that the samples possessed a nematic LC order. When the temperature was increased to 150 °C, the 2D‐WAXS pattern became circular, indicating that the LC molecules underwent a phase transition leading to a disordered arrangement of isotropic state. This was also confirmed by the POM characterization (Figure S3, Supporting Information).

The POM characterization of uniaxially aligned LCE film is shown in Figure 2c. The LCE film exhibited a longitudinal contraction and transverse expansion when heated above T_NI_, and the threaded‐texture gradually disappeared. When the temperature was lowered from 150 °C to 25 °C, the LCE returned to its original shape, showing a shape memory function. The deformation mechanism is shown in Figure S5 (Supporting Information). For the LCE cured without pre‐stretched, they formed polydomain structures. Mechanical tests were performed on the polydomain LCE, and the mechanical properties were found to be almost identical in all directions (Figure 2d), indicating that the LCE in the polydomain state had isotropic properties. For monodomain LCEs with a 300% pre‐stretch, stress‐strain curves (Figure 2e) and toughness (Figure 2f) were measured, showing smaller elongation at break and toughness due to macroscopic orientation, but significantly higher elastic modulus, providing a great incentive for mechanical work, compared to polydomain LCEs.

**Figure 3:**
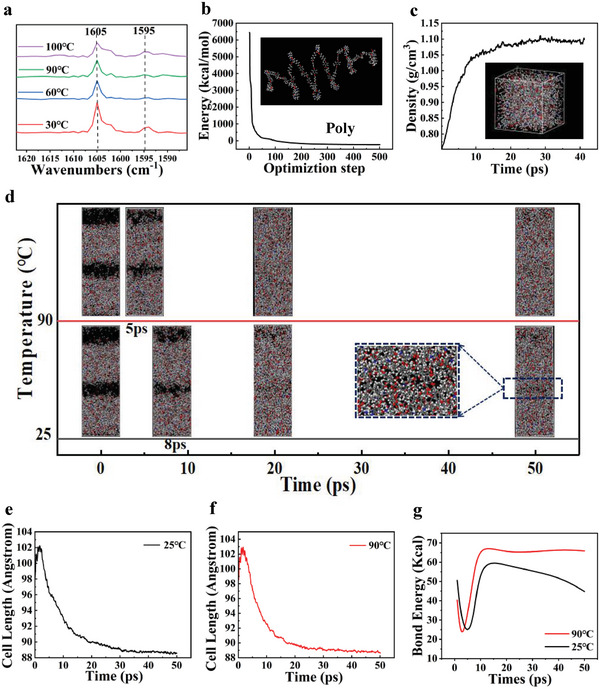
Characterization of the self‐healing performance of LCE. a) FTIR of the truncated surface of LCE. b) Energy minimal value model obtained by structural optimization. c) Variation of the box density and the final model obtained. d) LCE self‐healing process. e) Variation of box size during normal temperature self‐healing. f) Change in box size during high temperature self‐healing. g) Change in the number of hydrogen bonds during the self‐healing process.

To test whether the LCE has a self‐healing ability, two LCE films after the first‐stage cure were overlaid and fixed between two glass plates using a clamp to apply external pressure (Figure S6, Supporting Information). The assembly was heated at 90 °C in a vacuum‐drying oven for 12 h. Afterward, the sample was examined using scanning electron microscopy (SEM) to investigate the fractured surface of the composite LCE. The SEM image showed a good self‐healing condition (Figure S7, Supporting Information). To investigate the contribution of chemical bonding in the self‐healing process, the dynamic characteristics of hydrogen bonding at the truncated surface of the self‐healing LCE were investigated using variable temperature Fourier transform infrared spectroscopy (VT‐FTIR). **Figure** **3**a shows the FTIR spectra at the self‐healing LCE joints from 30 °C to 100 °C. The characteristic peaks at 1605 and 1595 cm^−1^ indicated the stretching vibration of the N─H group. The contribution of the two peaks gradually decreased with the increasing temperature. The hydrogen bonds dissociated releasing anchored N─H group to promote the bending vibration, which indicated that the hydrogen bonds played a strong bonding role in the self‐healing process.

In order to verify the accuracy of the test, molecular dynamics calculations were carried out uisng Materials Studio 2020. RM82 and 1,6‐hexanediamine were modeled (Figure S8, Supporting Information), homopolymerized according to the r = 0.9, and then the energy minima were obtained by structural optimization, and finally the polymer monomer model with energy minima was obtained (Figure 3b). A low‐density model (0.75 g cm^−2^) was built first to ensure that the structure is randomly distributed in the box. Then 10 000 steps of optimization are performed to complete the energy minimization of the box system (Figure S9, Supporting Information). Then, five cycles of annealing calculation (300–500 K) were performed to eliminate local high‐energy sites. After the optimization was completed, the molecules were subjected to molecular dynamics calculations for the normal pressure and temperature (NPT) system synthesis, after 50 ps of kinetic calculations at 25 °C to fully mix the molecules. The calculations were performed using a COMPASS II force field and a standard atmospheric pressure with temperature control by the nose and pressure control by Berendsen. The electrostatic energy and van der Waals were summed by Ewald and Atom based. A reasonable model was finally obtained (Figure 3c). A double‐layer extension model with 10 Å microcracks in the middle was created, and another 25 Å vacuum layer was created on the diffusion side due to periodic boundaries and directional diffusion (Figure S10, Supporting Information). The diffusion model was obtained by structural optimization. Molecular dynamics calculations were performed on the model at 25 °C and 90 °C to observe the conformational changes during the self‐healing process (Figure 3d). 8 ps entered the healing conformation at room temperature, while healing appeared earlier (5 ps) at high temperature, and the simulation results showed that the self‐healing effect could be significantly enhanced by increasing temperature (Movie S1, Supporting Information). Observing the box size (Figures 3e,f) and the number of hydrogen bonds (Figure 3g), both indicated that the elevated temperature facilitated molecular diffusion, allowing the structure to rapidly contact to form stable hydrogen bonds and then reached an equilibrium state. The ability of LCE to self‐heal, as demonstrated by simulations, was consistent with the experimental results, indicating that hydrogen bonding played a significant enhancing role in the self‐healing process.

Previously, mostly bio‐inspired embedded microcapsules were used, which were bonded by rupturing at the injury, thereby releasing the repair agent contained therein, which diffused to the crack and reacted. In these systems, a necessary condition for self‐healing is the contact of the defect with the repair agent embedded in the microcapsule, which triggers a polymerization reaction to repair the defect.^[^ ^43^ ^]^ Unlike previous bio‐inspired microcapsule systems that introduced repair agents into the material, in the amine‐acrylate LCE system, due to hydrogen bonding, there is no need for an external cross‐linking agent. The self‐healing process can be accelerated by warming up, which is a significant increase in the speed of self‐healing compared with the current LCE.^[^ ^18^ ^]^

### 2.2 Constitutive Model of LCE

Following the neo‐classical theory of nematic elastomers is developed to capture the coupled thermomechanical properties of LCE. We used the anisotropic parameter *r*, a director**n**and the deformation gradient **F**to describe the current configuration of the LCE relative to a stress‐free reference configuration, J=detF. δis introduced material parameter to reflect the extreme anisotropic degree, which depends material properties. κ means the propagating direction. Considering incompressible properties, the following free energy density is given by

(1) $$W=\frac{\mu }{2}\left(\mathrm{tr}\left(\ell_{n_{0}}F^{T}\ell_{n}^{-1}F\right)-3\right)+\frac{\kappa }{2}{\left(\ln J\right)}^{2}+C\frac{\delta -1}{{\left(r^{1/6}-\delta \right)}^{k}}$$

where μ is the shear modulus of the LCE, **n**_0_ is the unit vectors of the initial LCE, and

(2) $$\ell_{n}=r^{-1/3}\left(I+\left(r-1\right)n⊗n\right)$$

(3) $$\ell_{n_{0}}=r_{0}^{-1/3}\left(I+\left(r_{0}-1\right)n_{0}⊗n_{0}\right)$$

are the step‐length tensors in the current and reference configurations that collect the anisotropy parameter and the director. By solving the above equation by an extreme two‐step problem, the above energy function can be written as

(4) $$W=\frac{\mu }{2}\left(J^{-2/3}tr\left(F^{T}G^{-1}F\right)-3\right)+\frac{\kappa }{2}{\left(\ln J\right)}^{2}+C\frac{\delta -1}{{\left(r^{1/6}-\delta \right)}^{k}}$$

And the corresponding constitutive relation can be obtained in terms of the Cauchy stress by

(5) $$\sigma =\mu J^{-5/3}\left(F^{T}G^{-1}F-\frac{1}{3}\mathrm{tr}\left(G^{-1}C\right)I\right)+\kappa \ln JI$$

where *G* is introduced tensor of internal variables that follow an evolution law and shares the same principal basis as $B=F^{T}F$.

### 2.3 Design and Fabrication of the Origami‐Inspired Structure and Finite Element Simulation

**Figure 4:**
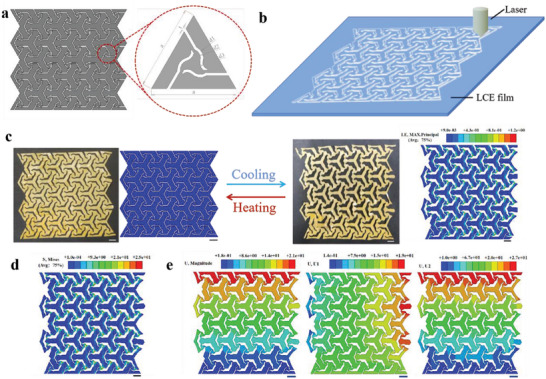
Experimental and finite element simulation of LCE paper‐cutting structure. a) Design of the negative Poisson's ratio paper‐cutting structure. b) Schematic diagram of laser cutting of negative Poisson's ratio LCE paper‐cutting structure. c) Experimental and simulation diagram of cold‐thermal cycle reversible deformation of pre‐programmed negative Poisson's ratio LCE paper‐cutting structure. d) Mises stress cloud of the pre‐programmed negative Poisson's ratio LCE shear structure with cool‐down reversible deformation. e) Equivalent displacement, X‐direction displacement, and Y‐direction displacement of the overall structure during the cooling‐reversal process of the pre‐programmed negative Poisson's ratio LCE shear structure. (Scale bar: 10 mm).

Inspired by the horseshoe‐shaped origami structure and the negative Poisson's ratio origami structure,^[^ ^3^, ^31^ ^]^ we embedded a concave horseshoe‐shaped microstructure inside a triangular module and constructed a variable deformable superstructure model using Solid Works with a length of L = 120 mm and a width of m = 104 mm (with a gap of t = 1 mm, d1 = 3.07 mm, d2 = 1.27 mm, d3 = 2.17 mm, and a = 20 mm) as shown in **Figure** **4**a.

The partially cured LCE film was cut into the designed variable deformable superstructure using a digital laser engraving machine (VLS 2.30, Universal Laser Systems, USA) (Figure 4b). The deformed superstructure was pre‐programmed for tensile deformation and fixed at 90 °C for 12 h to arrange the liquid crystal elements. The structure was then subjected to thermal cycling to achieve temperature‐driven reversible deformation (Figure 4c). The predicted deformation shape of the LCE superstructure by finite element analysis in Figure 4c matched well with the experimental results (Movie S2, Supporting Information). During the cooling and expansion process, the negative Poisson's ratio LCE origami structure with pre‐programmed deformation was driven by the rearrangement of liquid crystal elements due to the phase transition of the liquid crystal molecules inside the LCE. From the finite element simulation results, it can be seen that the maximum stress was mainly concentrated at the Y‐shaped cell connection at ≈29 MPa (Figure 4d). The U‐Magnitude was ≈21.2 mm, with a maximum X‐direction displacement of ≈19.3 mm and a maximum Y‐direction displacement of ≈27.3 mm (Figure 4e). Compared with the maximum displacement obtained from experimental testing (*U_eff_* = ≈20.8 mm, *U_x_* = ≈20.0 mm, *U_y_* = ≈27.9 mm), the results were in good agreement.

### 2.4 Design and Fabrication of 2D Lattice Superstructure and Finite Element Simulation

**Figure 5:**
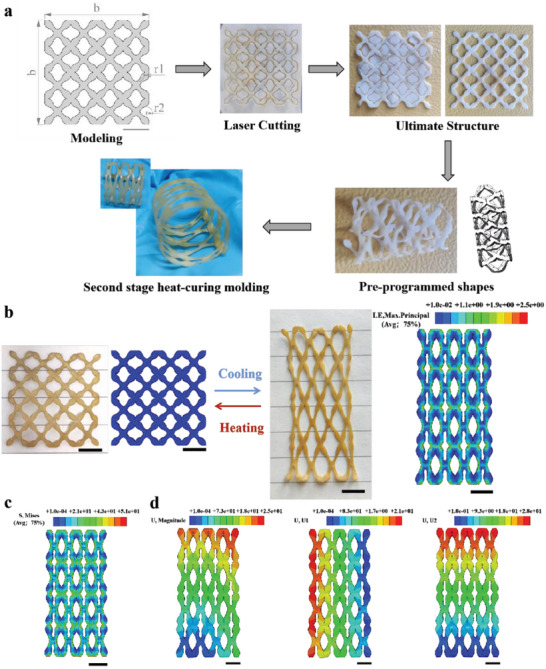
Experimental and finite element simulation of 2D lattice LCE superstructure. a) Diagram of the experimental process of preparing 2D lattice LCE superstructure and pre‐programmed sizing by laser cutting method. b) Experimental and simulation diagram of cold‐thermal cycle reversible deformation of pre‐programmed negative 2D lattice LCE superstructure. c) Mises stress cloud of pre‐programmed 2D lattice LCE superstructure with cool‐down reversible deformation. d) Equivalent displacement, X‐directional displacement and Y‐directional displacement of the overall structure during the cool‐down recovery of the pre‐programmed 2D lattice LCE superstructure. (Scale bar: 10 mm).

First, a 2D lattice LCE superstructure model with a side length of b = 40 mm was constructed using SolidWorks. The lantern‐shaped hollow structure has a maximum length and width of 7 mm, and all concave curved edges have a radius of r1 = r2 = 2.47 mm. The edge length of the X‐shaped cell is 10 mm. The pre‐programmed deformable superstructure was obtained using the same laser cutting and thermal curing method as described above. (**Figure** **5**a). Based on the shape memory function and temperature‐driven deformation characteristics of the LCE, when the temperature reached the T_NI_ of LCE or above, the liquid crystal molecules inside the 2D lattice LCE superstructure with pre‐stretched orientation underwent a phase transition, causing longitudinal shrinkage and transverse expansion, and then gradually recovered its programmed shape and can maintain elasticity at a maximum strain of 250% when the temperature decreased, which agrees well with the deformation shape predicted by finite element simulation (Figure 5b; Movies S3 and S4, Supporting Information). The finite element simulation results showed that the maximum stress was mainly concentrated at the X‐cell junction (≈51 MPa) due to the reorganization of liquid crystal elements. (Figure 5c); the U‐Magnitude was ≈25.2 mm, the maximum X‐direction displacement was ≈21.1 mm, and the maximum Y‐direction displacement was ≈28.6 mm (Figure 5d), which matched well with the maximum displacement measured by experimental testing (*U_eff_* = ≈25.9 mm, *U_x_* = ≈12.6 mm, *U_y_* = ≈33.1 mm).

## 3 Conclusion

In this study, we successfully synthesized a LCE with excellent mechanical properties (strain >400%), shape memory, and self‐healing capabilities by optimizing the amine/acrylate molar ratio (r = 0.9). The LCE was combined with two designed deformable structures (negative Poisson's ratio kirigami and 2D lattice structure), and the deformation process of the LCE superstructure was analyzed by constructing a large deformation constitutive model and using finite element simulation, validated by experiments, which overcomes the limitations of traditional deformable structures in substrate selection, amplifies the advantages of the superstructure deformation, and integrates the flexible self‐driven function of shape recovery and morphological reconfiguration after forming. 3D LCE superstructures using shape programming, building upon the previous research on 2D structures, were investigated, which showed great potential for applications in biomimetic artificial muscles, flexible temperature‐controlled switches (Figure S11, Supporting Information), and flexible heart stents (Figure S12, Supporting Information) (See Movies S5 and S6, Supporting Information for the deformation processes). These findings provide new ideas and methods for expanding the application of intelligent soft materials.

## 4 Experimental Section

### Materials and Preparation of LCE

1,4‐Bis‐[4‐(6‐acryloyloxyhexyloxy) benzoyloxy]−2‐methylbenzene (RM82) was purchased from Kindchem (Nanjing) Co., Ltd. 1,6‐Hexanediamine was purchased from TCI. N, N‐Dimethylformamide (DMF) and dichloromethane (DCM) was purchased from MACKLIN. Typically, 1 g of RM82 was dissolved in a mixed solvent of 0.3 g DMF and 1 g DCM, and melted at 80 °C to be completely dissolved. Then 0.1555 g of 1,6‐hexanediamine melted at a constant temperature of 60 °C was added and mixed to obtain a homogeneous precursor solution. The solution was then stirred for 30 min under sealed conditions at 60 °C. Next, the solution was poured into either a glass dish or a polytetrafluoroethylene mold for curing at 60 °C with a lid. For gel alignment, the LC gel was formed after 60 min and could be taken out of the dish and stretched to a strain of 200% and then was further cured at 90 °C for another 12 h (Scheme 1b).

### Material and Mechanical Characterizations of LCE

A polarizing microscope (POM BX51, Olympus, Japan) was used to observe the director alignment and nematic state of polydomain and monodomain LCEs. Wide‐angle X‐ray scattering (SAXSess mc2, Anton Paar) was used for characterizing the alignment of LCE. The glass and nematic‐isotropic transition temperatures were measured using differential scanning calorimetry (DSC) on a Mettler‐Toledo calorimeter (DSC3, CH). The samples were first ramped up and then cooled down in the temperature range of −50 °C to 150 °C at a rate of 10 °C min^−1^ to eliminate the effect of thermal history on their phase transition; the ramp‐up and ramp‐down processes were then recorded in the same manner, and the data obtained were normalized for comparison. Thermogravimetry analysis (TGA, JING YI GAO KE) was used to obtain the decomposition temperatures with a heating rate of 10 °C min^−1^ and at a nitrogen (N_2_) flow rate of 100 mL min^−1^. The mechanical properties with rectangular LCE samples mounted on the clamp were measured using dynamic mechanical analysis (DMA Q800, TA Instruments, USA). Fourier Transform Infrared spectroscopy (FTIR Nicolet iS10, Thermo Fisher, USA) was used to detect hydrogen bonds at self‐healing truncated surface joints.

### Finite Element Analyses

By constructing a large deformation intrinsic model of LCE materials and embedding the developed intrinsic model into the finite element software ABAQUS using the subroutine UMAT. The commercial software ABAQUS was used to perform the FEA, and the modified first‐order plane stress element (CPS6M) was used for discretization. Then numerical simulations of the deformation behavior of LCE driven by temperature and deformation were carried out using the finite element numerical model.

## Conflict of Interest

The authors declare no conflict of interest.

## Supporting information

Supporting Information

Supplemental Movie 1

Supplemental Movie 2

Supplemental Movie 3

Supplemental Movie 4

Supplemental Movie 5

Supplemental Movie 6

## Data Availability

The data that support the findings of this study are available from the corresponding author upon reasonable request.
