# Supplementary material for: Design and Characterization of Deformable Superstructures Based on Amine‐Acrylate Liquid Crystal Elastomers
Source: Adv Sci (Weinh). 2023 Nov 9;10(36):2303594. doi: 10.1002/advs.202303594 (PMC10754073; doi:10.1002/advs.202303594)
Supplement: Supplementary file 1 — Supporting Information [file ADVS-10-2303594-s003.pdf]

ADVANCED SCIENCE
Open Access

Supporting Information

for *Adv. Sci.*, DOI 10.1002/advs.202303594

Design and Characterization of Deformable Superstructures Based on Amine-Acrylate Liquid Crystal Elastomers

Fang Zhao, Yuzhan Li, Hong Gao*, Ran Tao*, Yiqi Mao*, Yu Chen, Sheng Zhou, Jianming Zhao and Dong Wang*

Supporting Information

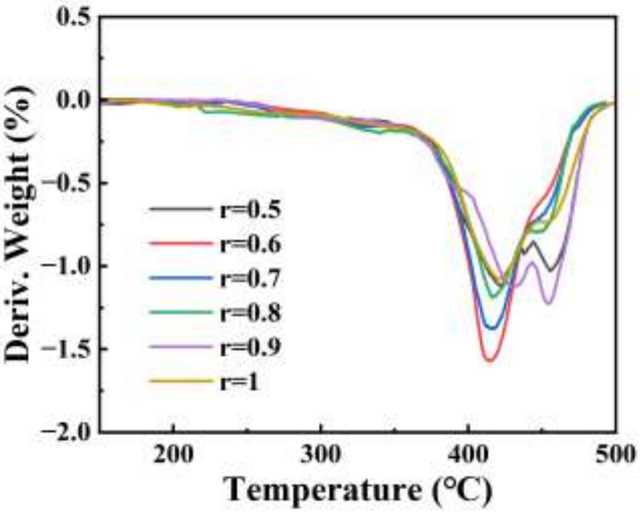

Figure S1. Derivative thermogravimetry (DTG) of LCEs with r = 0.5~1.

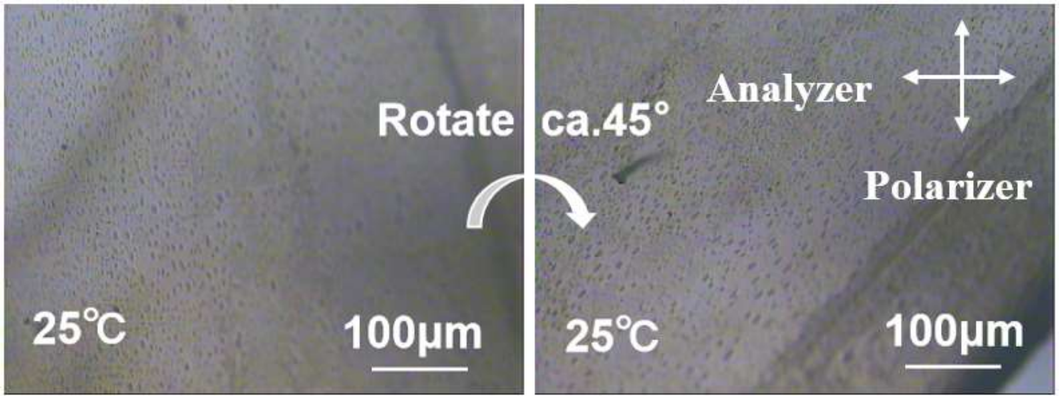

Figure S2. POM diagram before and after deflection of pre-stretched LCE $_{r\ =\ 0.8}$ film.

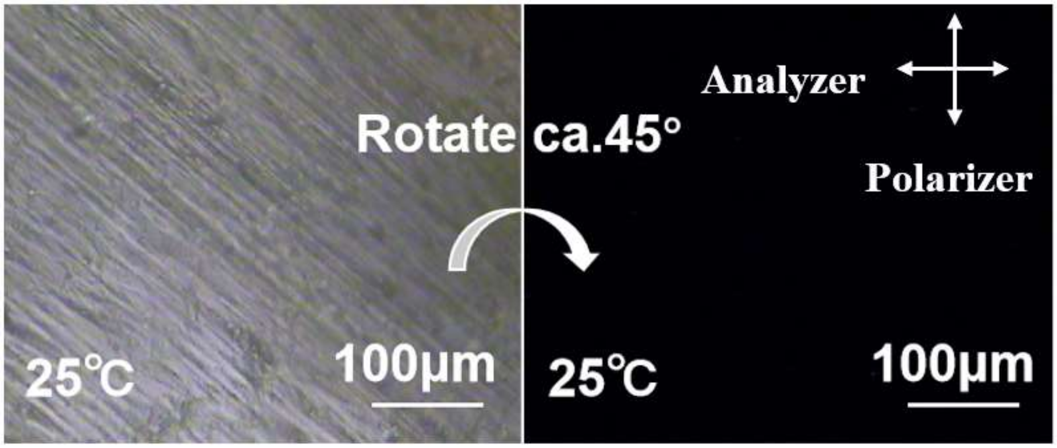

Figure S3. POM diagram before and after deflection of pre-stretched LCE $_{r = 0.9}$ film.

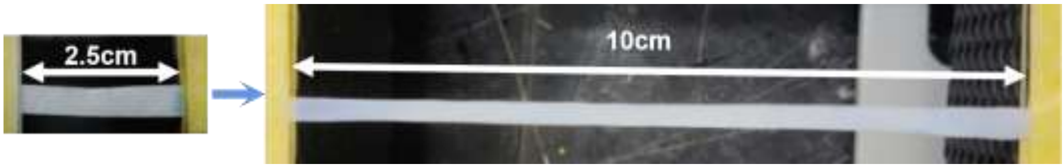

Figure S4. Partially cured LCE tensile deformation diagram.

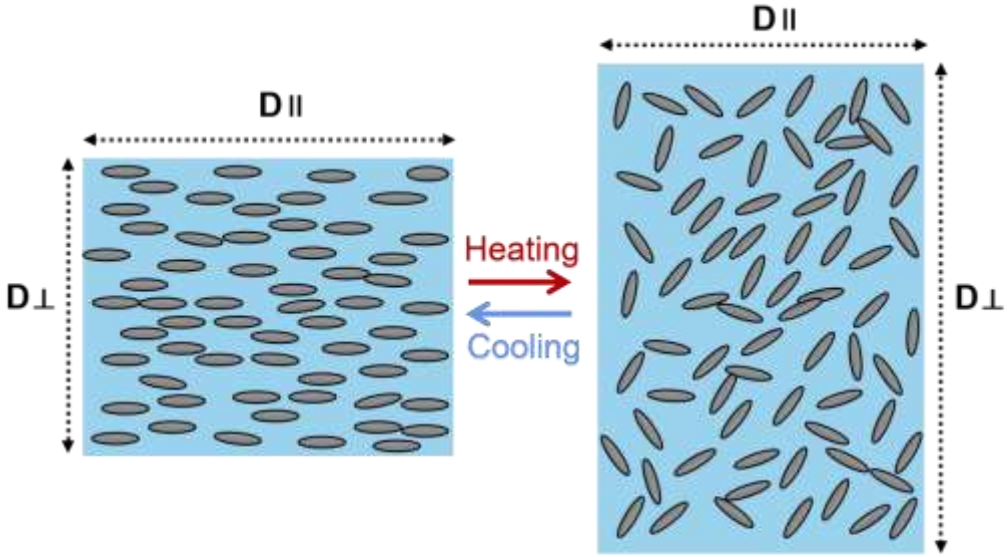

Figure S5. Reversible deformation mechanism of uniaxially aligned LCE films during heating and cooling cycles.

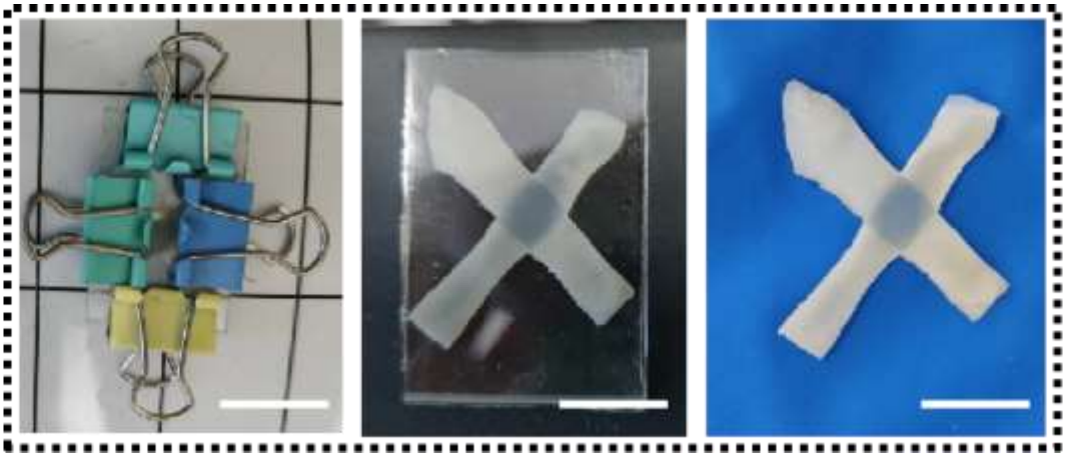

Figure S6. Self-healing experiment process diagram.

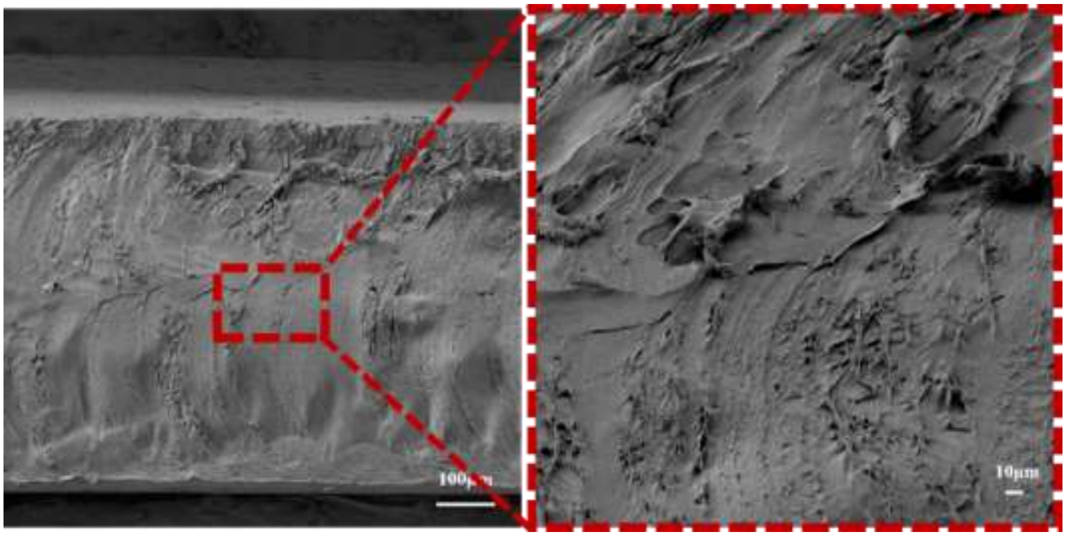

Figure S7. LCE self-healing cross-section SEM.

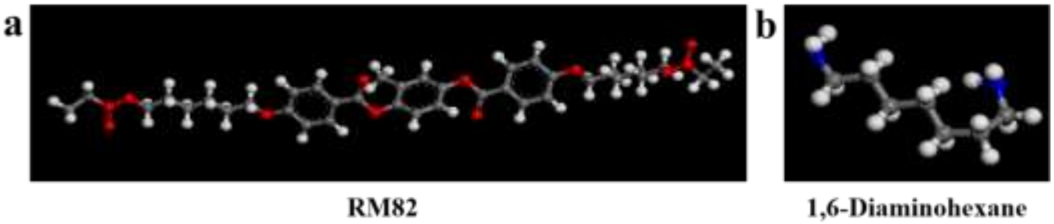

Figure S8. LCE polymer modeling. a) Reactant RM82 model. b) Reactant 1,6-hexanediamine model.

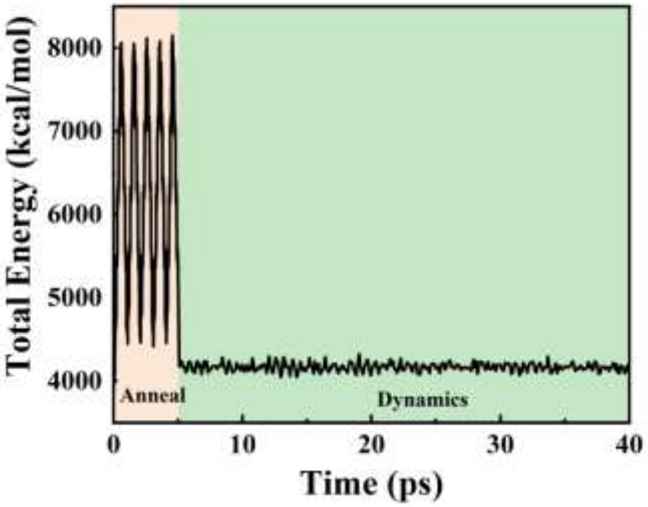

Figure S9. Energy changes during the optimization of the LCE polymer chain segment box model.

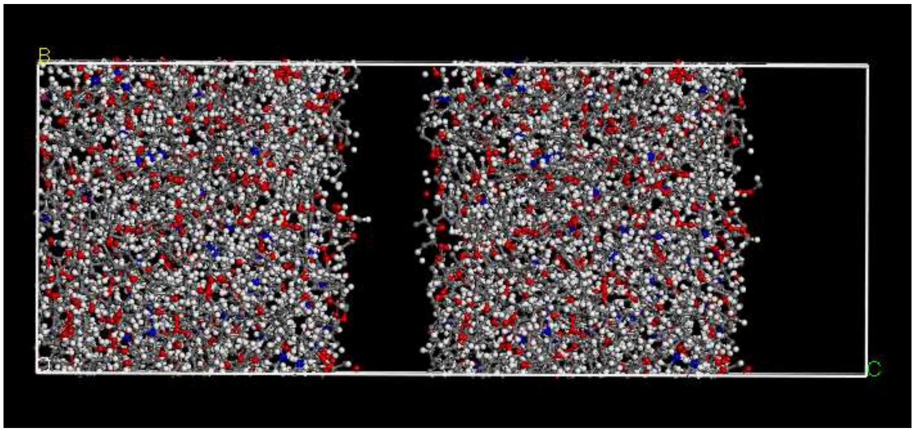

Figure S10. LCE self-healing model.

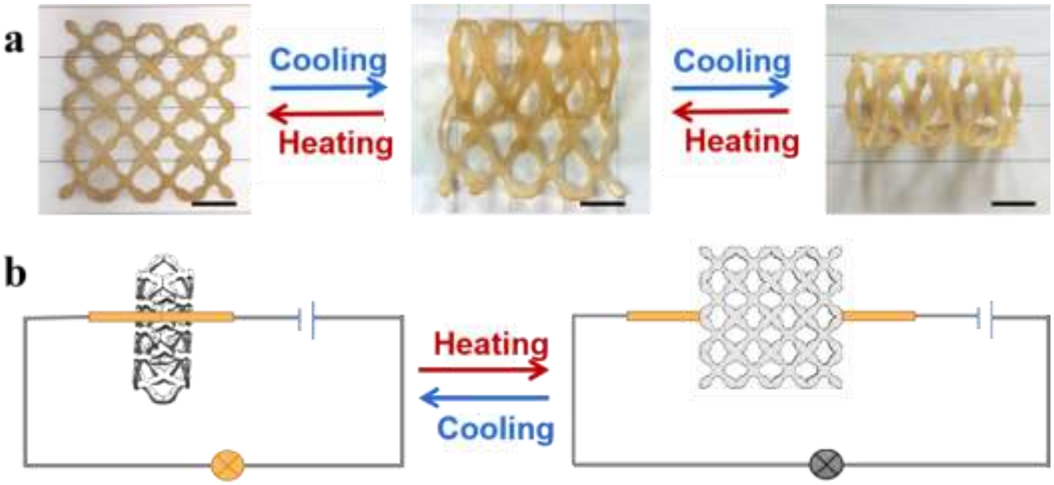

Figure S11. 3-dimensional deformation of 2D dot matrix LCE superstructure and application extension. a) Curl-unroll reversible deformation of temperature control system (scale bar: 10mm). b) Schematic diagram of temperature control switch.

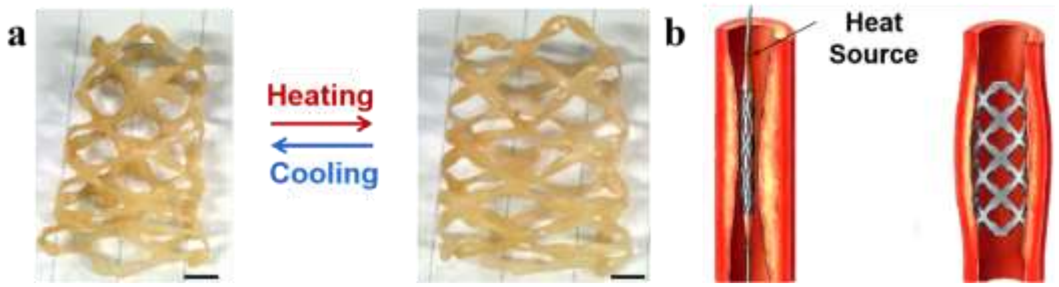

Figure S12. 3D deformation of 2D dot matrix LCE superstructure and application extension. a) Radial systolic-diastolic reversible deformation of temperature-controlled system (scale bar: 10mm). b) Schematic diagram of flexible.
